# Music Listening Among Postoperative Patients in the Intensive Care Unit: A Randomized Controlled Trial with Mixed-Methods Analysis

**DOI:** 10.1177/1178633717716455

**Published:** 2017-07-20

**Authors:** Nancy Ames, Rebecca Shuford, Li Yang, Brad Moriyama, Meredith Frey, Florencia Wilson, Thiruppavai Sundaramurthi, Danelle Gori, Andrew Mannes, Alexandra Ranucci, Deloris Koziol, Gwenyth R Wallen

**Affiliations:** 1 Nursing Department, Clinical Center, National Institutes of Health, Bethesda, MD, USA; 2 Pharmacy Department, Clinical Center, National Institutes of Health, Bethesda, MD, USA; 3 Department of Perioperative Medicine, Clinical Center, National Institutes of Health, Bethesda, MD, USA; 4 Biostatistics and Clinical Epidemiology Service, Clinical Center, National Institutes of Health, Bethesda, MD, USA

**Keywords:** Music, opioids, postoperative pain, ICU

## Abstract

**Background:**

Music listening may reduce the physiological, emotional, and mental effects of distress and anxiety. It is unclear whether music listening may reduce the amount of opioids used for pain management in critical care, postoperative patients or whether music may improve patient experience in the intensive care unit (ICU).

**Methods:**

A total of 41 surgical patients were randomized to either music listening or controlled non-music listening groups on ICU admission. Approximately 50-minute music listening interventions were offered 4 times per day (every 4-6 hours) during the 48 hours of patients’ ICU stays. Pain, distress, and anxiety scores were measured immediately before and after music listening or controlled resting periods. Total opioid intake was recorded every 24 hours and during each intervention.

**Results:**

There was no significant difference in pain, opioid intake, distress, or anxiety scores between the control and music listening groups during the first 4 time points of the study. However, a mixed modeling analysis examining the pre- and post-intervention scores at the first time point revealed a significant interaction in the Numeric Rating Scale (NRS) for pain between the music and the control groups (*P* = .037). The Numeric Rating Score decreased in the music group but remained stable in the control group. Following discharge from the ICU, the music group’s interviews were analyzed for themes.

**Conclusions:**

Despite the limited sample size, this study identified music listening as an appropriate intervention that improved patients’ post-intervention experience, according to patients’ self-report. Future mixed methods studies are needed to examine both qualitative patient perspectives and methodology to improve music listening in critical care units.

## Background

Music listening is a common, frequent, and universally enjoyed human endeavor. Listening to music, whether it is the classical notes of a Bach concerto or the hard beat of rap, produces powerful emotional, mental, and perhaps even spiritual feelings. These complex qualities and attributes of music are not easily explained.^1^ Admission to a critical care unit, especially after surgery, often produces fear of the unknown. This fear is intensified by the unfamiliar environment of critical care units which is often described as less than “patient-friendly.” The result, coined “intensive care unit (ICU) anxiety,” is often attributed to higher levels of noise and light pollution in critical care settings^2,3^; however, this ICU anxiety is truly multifactorial,^4^ often affected by postoperative pain. Clinical practice guidelines have recommended nonpharmacological interventions such as music therapy for the management of pain in critical care patients.^5^ Critical care nurses are in a unique position to investigate the efficacy of complementary, nonpharmacological interventions for reducing the burden of acute, postoperative pain and to examine methods for creating an ICU environment more conducive to healing.

Music has been used as a means of reducing patient anxiety before, during, and after surgery^6–9^ and has many advantages including its low cost, high feasibility, and low risk of adverse effects. Music is believed to be part of the neurological reward pathway, and stimulation of the mesolimbic system by music has been demonstrated by Menon and Levitin^10^ using functional magnetic resonance imaging (fMRI). Music may divert the listener’s attention away from an unpleasant episode, such as pain, and instead focus attention on the music listening experience.^1^ In a Cochrane Review, music listening experiences were reported to provide peacefulness and tranquility to hospitalized patients during a time of great distress; for example, patients undergoing treatment for coronary heart disease.^11^ The purpose of this randomized, controlled trial was to determine the efficacy of music listening as a means of reducing pain, anxiety, distress, and opioid use in patients admitted to a critical care unit following surgery.

## Methods

### Study design

This study was a randomized, controlled trial that evaluated the effects of music listening on eligible surgical patients’ opioid use and self-reported pain, distress, and anxiety. Participants were consented preoperatively, but randomized postoperatively to either a music listening or a control group. The control group received standard postoperative care supplemented by an approximately 50-minute period of rest instituted to match the 50-minute music listening period of the experimental group.

### Recruitment and eligibility

This study was conducted over a period of 18 months (August 2011 to February 2013). Study approval was obtained from the National Cancer Institute’s intramural Institutional Review Board (NCT01409044, ClinicalTrials.gov). The principal investigator screened and evaluated lists of surgical patients admitted to the NIH Clinical Center (CC) on a weekly basis, contacted eligible patients preoperatively, and invited them to participate. Adult (18 years of age or older) surgical patients at the NIH CC who understood and spoke English or Spanish, with an anticipated postoperative ICU stay of 24 to 48 hours, and anticipated use of a patient-controlled analgesia (PCA) device for postoperative pain management were considered eligible. Eligible patients were consented prior to surgery and data collection by the principal investigator or a trained associate investigator. Patients were not eligible for enrollment if they were scheduled for a neurological procedure, were visually or hearing impaired, or met criteria for severe anxiety. The General Anxiety Disorder (GAD-7) questionnaire was used to screen for severe anxiety,^12^ as music listening was not anticipated to mitigate its effects.^13^ Therefore, patients with a GAD-7 score of 15 or higher (15-21 = severe anxiety) were excluded.

### Study procedures

#### Preoperative

Participant demographic data were recorded and patients were educated on the use of the PCA device. In addition to the GAD-7, preoperative anxiety was measured using the State-Trait Anxiety Inventory (STAI) and the Emotional Thermometers (ETs) with permission from both authors.^14–16^ The STAI was only used preoperatively. The STAI is a 2-part, 40-question survey developed by Charles D. Spielberger et al.^16^ The initial half of the survey establishes participant “state,” an anxiety score at the point of survey administration.^17,18^ The final 20 questions examine subject “trait,” a measure that accounts for “long-standing quality” of anxiety.^17^ The STAI scoring followed the guidelines provided by Spielberger et al.^19^ Pain was measured using the visual analog scale (VAS) and Numerical Rating Scale (NRS).^20,21^ In completing the VAS, patients indicated the severity of their pain on a scale with markings from 0 to 100 mm.^20^ A marking of 0 mm represented no pain and 100 mm represented the most severe pain.^20^ Patients verbally reported their pain on a scale from 0 to 10 to complete the NRS.^21^ The ETs score anxiety and distress on a scale of 0 to 10.^22^ All of these measures were previously validated in many other patient populations and are provided in the supplementary appendix.^23–26^ Opiates, benzodiazepines, and other drugs that could effect patients’ tolerance to pain medication were recorded 24 hours before surgery and during the postoperative period.

#### Postoperative

The critical care unit at the NIH CC is a multidisciplinary, 18-bed medical-surgical unit that admits patients requiring intensive monitoring because of critical illness; all admitted patients are participants in a research-related protocol, directly related to their illness. The critical care unit is staffed by critical care nurses, respiratory therapists, and intensivists. The surgical patients are jointly managed by surgeons and intensivists. Acute pain management techniques involve intravenous opioids and/or peripheral or epidural PCA. Postoperative management commonly involves epidural catheters and PCA devices. Nurses usually care for a maximum of 2 patients. On ICU admission post-surgery, subjects were randomized to either music listening or control groups. Randomization criteria included the ability to verbally answer questions regarding pain and anxiety. Patients who were intubated were not randomized. In addition, an active order for pain medication using a PCA device was required. Randomization was conducted using opaque, sealed envelopes with group designations that had been prepared previously by the statistician using a computer-generated, permuted block randomization schema. The research staff was blind to the contents of the envelopes. Once the patient was randomized, the appropriate bedside booklet was provided that listed all the measures and time points for ease of documentation by the nurses in the ICU. Prior to beginning the study, this booklet and all the study measures were reviewed by the ICU staff.

The music listening group was exposed to the MusiCure Dreams album (Gefion Records, Copenhagen, Denmark) using noise-canceling headphones for approximately 50-minute intervention(s) during their ICU stay.^27^ Patients were instructed to listen to the entire selection. The music from this company was selected for use in medical settings and had been used in similarly designed studies.^28,29^ The control group participants experienced approximately 50-minute quiet resting periods at the same frequency as music listening participants. No restrictions were placed on nursing care or medical care during interventions. However, each nurse was asked to perform a “settling routine” before the intervention to prepare the surgical patient. Repositioninng, mouth care and dimming lights were part of this routine. Both groups received this settling routine. Initially, music listening patients received a maximum of 3 sessions each day, at approximately 8-hour intervals. Music intervention periods did not occur more often than within 4 hours of the previous session. Other than these limitations, patients could ask to listen to the music at any time during their 48-hour ICU stay. After enrolling the first 3 patients to the protocol, music interventions were increased to every 4 to 6 hours to maximize the frequency of interventions among patients who were discharged earlier than 48 hours. The control group also followed these guidelines.

Before and after each resting period or music intervention, anxiety and distress were measured using the ETs, and postoperative pain was determined using both the NRS and VAS. Opioid intake was measured using PCA devices (Gemstar; Hospira, Lake Forest, IL, USA) during the entire ICU stay. Study participants were categorized by PCA opioid administration route, with epidural and intravenous PCA routes measured separately, in units of micrograms of fentanyl or milligrams of morphine. All opioids were calculated for each patient, including both PCA-delivered and additional doses of analgesia. All nonepidural opioids were converted to milligrams of morphine using a standard equianalgesic table calculation (Supplementary Appendix B).^30^

Opioid administration was recorded during each intervention. Music or resting interventions were conducted for a maximum of 48 hours. Patients received a variable number of interventions (1-8 sessions) depending on the length of their ICU stay and their compliance. Interviews were conducted by the study team after ICU discharge to assess ICU and music study experiences.

### Data analysis

The study is a randomized, controlled trial with 2 groups: music listening and quiet resting control group. Descriptive statistics were performed on all study variables at each time point. Bivariate relationships of the main outcomes (pain, anxiety, and distress) were examined by correlations (Spearman correlation coefficient) for all cases. The Fisher exact test, *t* test, 1-way analysis of variance, and the Mann-Whitney *U* test were used to examine the differences in demographic, clinical variables, and the main outcomes between groups. Unless otherwise noted, data are presented as mean values and their standard deviations.

Linear mixed models were used to examine whether there was a change in pain, anxiety, or distress between groups during the first 4 interventions. The difference scores were calculated by subtracting the pre-intervention score from the post-intervention score for each intervention. A separate set of mixed models examined whether a statistical difference existed between the 2 groups, prior to and following the first intervention. Intravenous and epidural PCA opioid intakes were compared between music and control groups in the first and second 24-hour periods. A restricted maximum likelihood procedure was used for model parameter estimation. Akaike information criterion (AIC) and Bayesian information criterion (BIC) were used to compare models.^31^ In linear mixed models, the response depends on both population-level parameters (fixed effects) and subject-specific random effects. Linear mixed modeling does not require the same number of observations in each subject. It also does not require that all the measurements be taken at the same time. In this repeated measures study, missing data can be predicted by the model. For mixed models, the estimates (“least square means”) and the associated standard error of the estimates with the *P* value are reported. All data analyses were done using IBM SPSS statistics version 21 (IBM Corp., Armonk, NY, USA) and SAS version 9.3 (SAS Institute, Cary, NC, USA). A *P* value of .05 or less was considered statistically significant.

Qualitative analysis was performed from the transcribed interviews of the music group. A semi-structured interview guide was used with each participant (Supplementary Appendix C). Independent thematic analysis was conducted by 3 research team members. This was followed by 2 consensus building meetings on the initial themes generated. These themes were placed in NVivo (Version 10, Burlington, MA, USA) to better visualize theme patterns and understand the prevalence of each theme. These themes were reverified by a team member (N.A.) with clinical ICU experience to further establish credibility and reproducibility of the generated themes.

## Results

### Sample demographics

**Figure 1.**
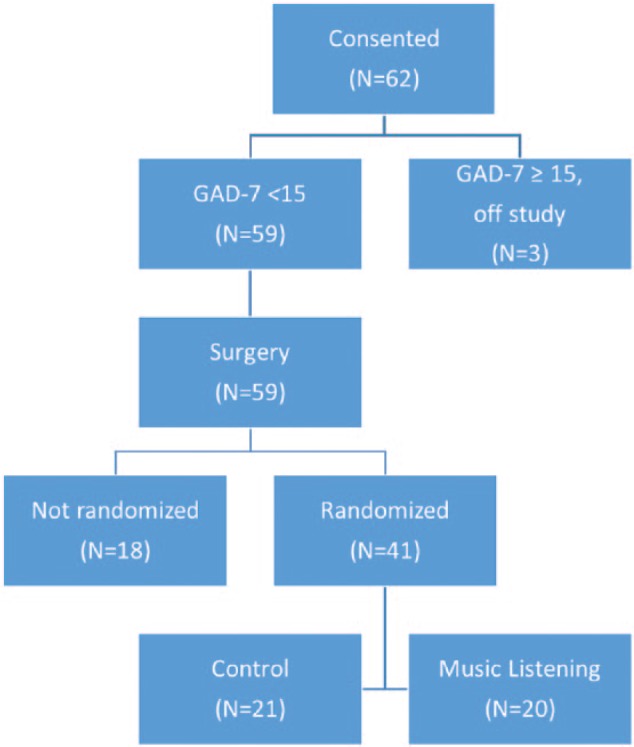
Study flow chart. Progression of study participants through consent and randomization.

A total of 62 patients were preoperatively screened and consented to this protocol. All 62 patients completed a screening GAD-7 questionnaire. Three patients failed to qualify due to a high score on the GAD, indicating severe anxiety. Table 1 describes demographics and comorbidities of the total sample (n = 59) comparing the music, control, and non-randomized groups. The non-randomized group constituted those patients who were consented but did not meet the criteria for randomization after surgery. Eighteen patients did not meet the eligibility criteria for randomization. The primary reasons that patients were not randomized were lack of participant admittance to the ICU (n = 7) and intubation for more than 4 hours following surgery (n = 7). The other 4 were not randomized due to lack of PCA orders (n = 3) and 1 remaining patient’s surgery was canceled and not rescheduled. Figure 1 is the study flow diagram for participants.

**Table 1.** Sample characteristics, N = 59.

Variables		Control	Music	NR	Total	*P* value
***Demographics***						
Gender	Males	12	10	10	32	.897
	Females	9	10	8	27	
Race	White	18	16	15	49	.903
	Black/AA	3	3	2	8	
	Asian	0	1	0	1	
	Unknown	0	0	1	1	
Age, y	Mean (SD)	52.95 (15.09)	52.45 (13.48)	57.28 (10.10)	54.10 (13.14)	.473 ^a^
	Minimum/maximum	27–73	29–83	32–73	27–83	
	Interquartile range	23.50	20.8	13.25	19.00	
Surgery	Nephrectomy	8	9	7	24	.250
	Thoracotomy/lobectomy	5	4	1	10	
	Abdominal surgery^b^	4	7	4	15	
	Adrenalectomy	1	0	2	3	
	Other^c^	3	0	4	7	
***Comorbidities***						
Hypertension		10	10	10	30	.898
Alcohol use		17	12	9	38	.123
Prior opiate use		4	2	4	10	.627
Substance abuse		0	2	1	3	.399
Smoking		4	2	1	7	.540
***Postoperative analgesia***						
Type of PCA^d^	Intravenous	9	11		20	.719
	Epidural	10	8			
	IV and epidural	2	1			
PCA drug^e^	Epidural fentanyl	11	9		20	.552
	Hydromorphone	6	7		13	
	Nonepidural fentanyl	1	3		4	
	Morphine	3	1		4	
ON-Q pump^f^	Bupivacaine 0.25%	4	4		8	.627
	Bupivacaine 0.50%	2	4		6	

Abbreviations: AA, African American; ICU, intensive care unit; NR, not randomized; NA, not applicable; PCA, patient-controlled analgesia.

Table 1 explores the sample characteristics by examining demographics, comorbidities, and postoperative analgesic use. This table is separated into the music, control, and non-randomized groups. The non-randomized group comprised those patients who were consented but did not meet the criteria for randomization after surgery. Three patients scored 16 on the General Anxiety Disorder study and were not eligible for inclusion. These 3 patients are not included in the data analysis.

a All comparisons used the Fisher exact test except the variable “age” that was calculated with a 1-way analysis of variance.

b Abdominal surgery includes abdominal resections, small bowel resection, pancreatectomies, gastrectomies, and abdominal perineal resection.

c Other category includes adrenalectomy that was performed laproscopically, ileal conduits/cystectomies, and exploratory laparotomy. One patient had surgery canceled. This patient is included in the other (not randomized) group.

d Three patients had both intravenous and epidural PCAs during their ICU stay.

e PCA drug initiated in the ICU. If the patient had more than 1 drug in the ICU, only the first drug is reflected here.

f ON-Q pump is a type of elastomeric balls that can be adjusted to deliver a prescribed amount of local analgesic. These pumps deliver bupivacaine directly into the incision using elastomeric balls that can be adjusted to deliver a prescribed amount of local analgesic. The concentration of the bupivacaine is either 0.25% (2.5 mg/mL) or 0.50% (5 mg/mL). This concentration is the concentration that was initiated in the ICU in the first 24 hours.

Forty-one patients were randomized postoperatively (music intervention group n = 20, control group n = 21). There were no significant differences in any of the characteristics among the music and the control group nor was there any significant difference among those 2 study groups and the participants who failed to randomize. Participants ranged from 27 to 83 years of age, with an average age of 54 years. Most of the study participants were white (83%) and had approximately 16 or more years of education (55%, n = 55). Other comorbidities, including diabetes mellitus, chronic obstructive pulmonary disease/emphysema, fluid and electrolyte disturbances, and cardiac arrhythmias were rare in the sample. There were a variety of admitting diagnoses, the vast majority secondary to cancer. Von Hippel-Lindau disease (n = 12), Birt-Hogg-Dubé (n = 3), and renal cell carcinoma (n = 3) were the 2 most common diagnoses in the sample. Other forms of cancer including rectal, renal, bladder, and lung cancers were also present in this cohort. A nephrectomy was the most common type of surgery.

All 59 patients completed the STAI, baseline ETs, and pain scales. The mean score for the state anxiety inventory was 34.8 (11.0) and the trait was 30.6 (9.4). The mean score of the GAD (n = 59) was 4.1 (2.9). The ETs had low mean scores at baseline with mean ET Anxiety of 2.6 (2.3) and the mean ET Distress of 1.8 (2.0). The mean VAS was 5.2 (10.0) mm and the mean NRS was 0.63 (1.31). The preoperative VAS and NRS pain scales were found to be strongly correlated (0.886; *P* = .01). The other screening tools were all strongly correlated with both State and Trait Anxiety with a *P* value at the .01 level, the ET Distress (State: 0.447; Trait: 0.461), and ET Anxiety (State: 0.658; Trait: 0.430), as well as the GAD-7 (State: 0.516; Trait: 0.442).

There was no significant difference in any of the measures performed preoperatively at baseline between the music and control groups (Table 2). In addition, Table 2 includes the pre-intervention scores compared with the scores taken before the first intervention after surgery and randomization.

**Table 2.** Baseline and pre-intervention measurements.

Variable	Mean (SD)		Median		*P* value^a^	N
	Music	Control	Music	Control		
State Anxiety	34.60 (12.15)	35.33 (12.22)	31.50	35.00	.764	41
Trait Anxiety	28.60 (6.80)	32.48 (12.10)	27.00	29.00	.448	41
GAD-7	3.75 (2.59)	4.62 (3.41)	3.00	4.00	.545	41
NRS baseline	0.80 (1.57)	0.67 (1.39)	0.00	0.00	.692	41
NRS pre-intervention	5.05 (3.01)	3.67 (2.18)	5.00	4.00	.093	41
VAS baseline	5.01 (8.40)	6.06 (11.76)	2.05	0.00	.570	41
VAS pre-intervention	49.00 (30.91)	29.85 (24.04)	55.00	22.00	.074	40
ET Distress baseline	2.00 (2.43)	1.52 (1.69)	1.00	1.00	.677	41
ET Distress pre-intervention	3.70 (3.39)	2.10 (2.20)	3.00	2.00	.163	40
ET Anxiety baseline	2.48 (2.29)	3.14 (2.67)	2.00	3.00	.399	41
ET Anxiety pre-intervention	3.05 (2.80)	1.80 (1.99)	3.50	1.50	.165	40

Abbreviations: ET, Emotional Thermometer; GAD-7, Generalized Anxiety Disorder 7; N, sample size; VAS, visual analog scale.

Table 2 compares the baseline measures collected prior to surgery after consent with the same measures collected pre-intervention. Pre-intervention (time point 1) scores were obtained after surgery and randomization on admission to the intensive care unit and prior to any intervention.

GAD-7,^12^ Emotional Thermometers (ET Distress and Anxiety),^22^ State Anxiety and Trait Anxiety from the State-Trait Anxiety Inventory, Spielberger et al.^16^ Mann-Whitney *U* test was used to compare the music and control with mean and median scores reported.

a Mann-Whitney *U* test.

### Preoperative data

Medications administered preoperatively that affected opiate tolerance were collected from all study participants. Table 1 lists prior opiate use comparing the music and control groups. Besides anxiolytics and medications ordered for sleep, very few patients were ordered other medications prior to surgery. The most common class of drugs was sedatives. In total, 13 patients (5 patients in the music group and 8 in the control group) were taking sedatives with the majority taking zolpidem. Other comorbidities including substance abuse, alcohol use, smoking, and hypertension were compared between the music and control groups (Table 1).

### Postoperative data

All of the 41 randomized patients started at least 1 intervention, and 66% completed at least 4 interventions. One patient started the intervention but refused to listen to the music. Two other patients in the music group did not listen to the entire first selection. This attrition was caused by patients transferring out of the ICU early, requesting not to listen to music for numerous other clinical reasons. The nurses were instructed to offer the interventions every 4 to 6 hours for the first 48 hours. Most of the interventions were offered every 6 hours, but the requirement was to complete at least 4 interventions per day with at least 4 hours between interventions.

Three opioids were used in this study for PCA delivery: fentanyl, morphine, and hydromorphone. Table 1 displays the type of PCA ordered between the music and control groups. During the first 24 hours of the study, 20 patients (48%) received epidurals with fentanyl used as the opioid. In all the 20 patients, bupivacaine in concentrations of either 0.0625% or 0.1250% was coadministered in the same fluid with the fentanyl. One patient received only bupivacaine (0.125%) in the epidural and received hydromorphone via intravenous PCA. Orders for the intravenous PCAs were written by surgeons and nurse practitioners, whereas epidural orders were prescribed and managed by an anesthesiologist or nurse anesthetist. Because the CC is both a research and teaching institution, many surgeons prescribed postoperative opioids, and the practitioners in the anesthesia department routinely ordered epidural medications postoperatively. In addition to the PCA opioids, data on other drugs administered during the study were collected. Extra doses of hydromorphone, outside of the PCA, were administered to 9 patients (6 in the music group, 3 in the control group) during the first 24 hours. Only 1 patient was administered hydromorphone in the second 24 hours. Other opiates administered in the first 24 hours included 3 patients who had fentanyl (2 patients in the music group, 1 in the control group). One patient in the music group had 1 dose of Demerol. Ketorolac, a nonsteroidal anti-inflammatory drug, was administered to 9 patients (4 in the music group, 5 in the control group) in the first 24 hours and 7 patients (3 in the music group, 4 in the control group) in the second 24 hours. In the first 24 hours, 14 patients had ON-Q pumps (Kimberly Clark Health Care Company, Irvine, CA, USA). These pumps deliver bupivacaine directly into the incision using elastomeric balls that can be adjusted to deliver a prescribed amount of local analgesic. Comparing patients with an ON-Q pump to those without, there was no significant difference in total intravenous PCA opioids used (patients with epidurals did not have ON-Q pumps).

**Figure 2.**
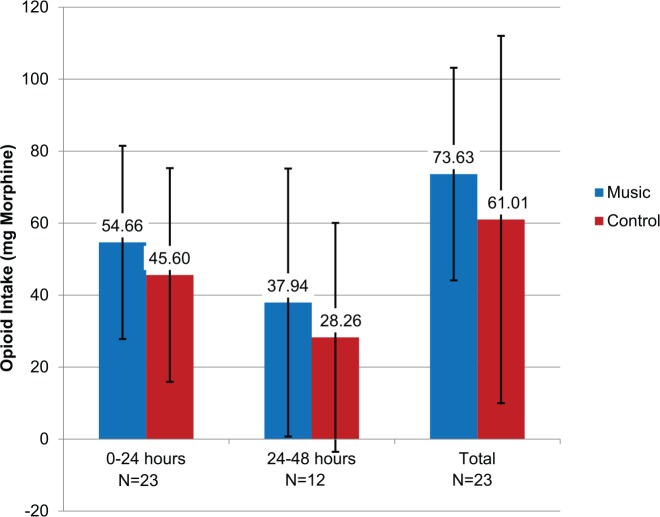
Intravenous patient-controlled analgesia opioid use during intensive care unit (ICU) stay. Mean (arithmetic) doses of opioids taken during the stay in the ICU. Doses are reported as morphine. These doses include the continuous rate of the morphine as well as the patient-selected boluses. There was no significant difference between the music and control at any time point or for overall doses. If the opioid was delivered intravenously, then the opioid was converted to morphine. The conversion follows a standard equianalgesic table (see Supplementary Appendix B).^30^

Figure 2 compares the arithmetic mean of intravenous opioids delivered to the music and control groups. During the study period, 12 patients in the music group required a mean of 73.63 (SD: 29.54) mg of morphine/morphine equivalent drugs, and the control group opioid use demonstrated very little difference with 11 patients using a mean of 61.01 (SD: 51.03) mg of morphine/morphine equivalent drugs (*P* = .471, independent sample *t* test). Similarly, within the first 24-hour period, average intake of opioids administered intravenously again demonstrated little difference in the music group (54.66, SD: 26.86) mg as compared with control subjects (45.60, SD: 29.70) mg (*P* = .451, independent sample *t* test). The same trend was observed within the second 24-hour period of ICU admission for music (37.94, SD: 37.24) mg and control (28.26, SD: 31.79) mg groups (*P* = .639, independent sample *t* test).

**Figure 3.**
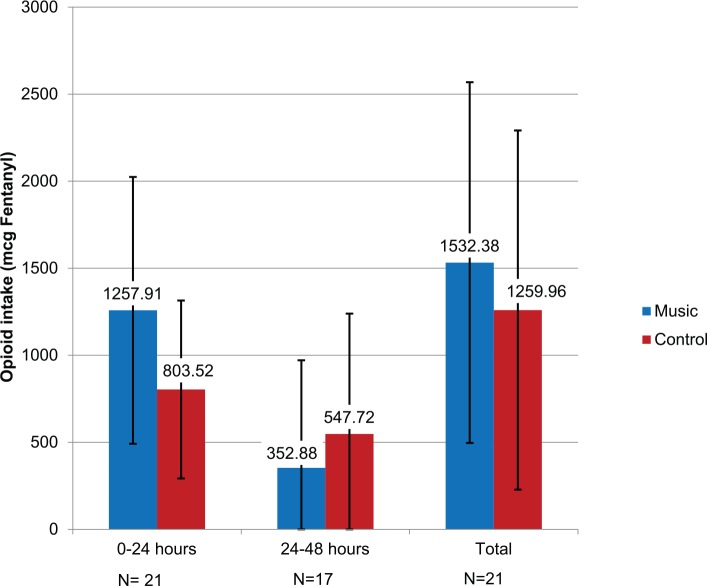
Epidural patient-controlled analgesia opioid intake during intensive care unit (ICU) stay. Mean (arithmetic) doses of fentanyl taken during the stay in the ICU. All doses were delivered using epidural catheters. These doses include the continuous rate of the morphine as well as the patient-selected boluses. There was no significant difference between the music and control at any time point or for overall doses. Sample size = 21.

Figure 3 displays the same comparisons but with the epidural dosing of fentanyl. During the entire study period, 9 patients in the music group used a mean of 1532.38 (SD: 1036.22) mcg of fentanyl. The mean epidural use for the control group was 1259.96 (SD: 1031.82) mcg of fentanyl (*P* = .557, independent sample *t* test). However, none of these values were statistically significant. In the first 24-hour period, average intake of opioids in epidural PCA was higher in the music group (1257.91, SD: 766.35 mcg) as compared with control subjects (803.52, SD: 511.14 mcg), but not significant (*P* = .119, independent sample *t* test). The reverse was observed within the second 24-hour period, in which the music group had lower epidural opioid intake (352.88, 618.30 mcg) as compared with control subjects (547.72, SD: 691.71 mcg). Again, this value was not significant (*P* = .560, independent sample *t* test).

**Figure 4.**
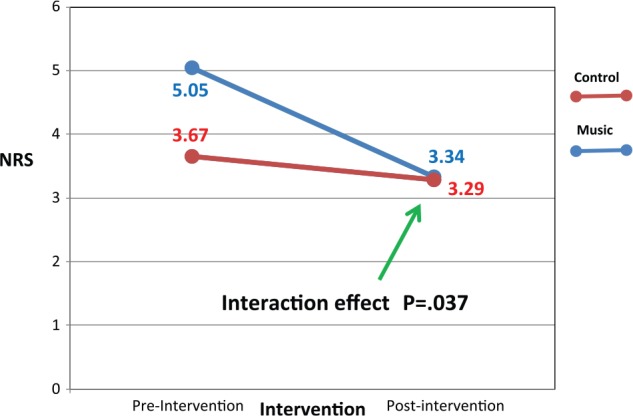
Estimated marginal mean Numeric Rating Score before and after first intervention. Prescore (music = 20; control = 21); postscore (music = 17; control = 20). This figure displays the results of the mixed model for repeated measures for the Numeric Rating Scale (NRS) least square means for time point 1. There was a significant interaction effect between time point 1 pre/post NRS scores and the music group. Although the control group’s NRS scores remain relatively stable, the music group’s pain scores decreased. Although both NRS scores decreased, the music group’s decrease is more pronounced than the control.

Linear mixed modeling showed no significant difference between the music and control groups in pain, distress, and anxiety difference scores in the first 4 interventions. The differences in the model-estimated mean values and standard errors are presented in Table 3 for pain, anxiety, and distress. Most of the scores are negative, demonstrating that the pre-intervention scores are usually higher than the post-intervention scores. However, examining the pre- and post-intervention scores at the first time point, there was a significant interaction in the NRS between the music versus the control groups (β = 1.334, SE = 0.614, *P* = .037) (Figure 4). The calculated Cohen *d* = 0.34. Unstructured covariance structure was selected based on smaller AIC and BIC values. The changes from pre- to post-intervention are significantly different between the music and control groups. The music group changed from an estimated marginal (least square) mean score of 5.05 (SE: 0.53) pre-intervention to 3.34 (SE: 0.56) post-intervention, whereas the control group mean remained relatively constant at 3.67 (SE: 0.52) pre-intervention to 3.29 (SE: 0.53) post-intervention. Table 4 displays the estimated marginal (least square) means for this first intervention.

**Table 3.** Differences in model estimated mean (standard error) for pain, distress, and anxiety scores.

Variable	Group	Differences (post-intervention − pre-intervention) in model estimated mean			
		1	2	3	4
NRS (0-10)	Music	−1.50 (0.36)	0.15 (0.35)	−0.03 (0.39)	−0.18 (0.43)
	Control	−0.40 (0.33)	−0.31 (0.33)	0.35 (0.35)	−0.80 (0.37)
VAS (0-100 mm)	Music	−13.05 (3.80)	0.29 (3.70)	0.92 (4.05)	−1.89 (4.53)
	Control	−7.82 (3.60)	−2.96 (3.51)	3.93 (3.70)	−8.83 (4.05)
ET-A (0-10)	Music	−0.85 (0.53)	−0.46 (0.41)	−0.08 (0.25)	−0.69 (0.53)
	Control	0.07 (0.50)	−0.18 (0.39)	−0.07 (0.23)	0.46 (0.47)
ER-D (0-10)	Music	−1.35 (0.54)	−0.71 (0.25)	−0.30 (0.28)	−0.37 (0.34)
	Control	−0.12 (0.51)	0.004 (0.23)	−0.28 (0.25)	−0.19 (0.29)

Abbreviations: ET-A: Emotional Thermometer for Anxiety; ET-D: Emotional Thermometer for Distress; VAS, visual analog scale.

Table 3 displays the 4 major time points (interventions) of the study and the differences in the linear mixed model estimated means (least square means). The scale and minimum and maximum score are given. The linear mixed model means takes into account missing data at the various intervention points, unlike an arithmetic mean. A negative value means that the pre-intervention value was higher than the post-intervention. Most of the scores are negative in both control and music groups which demonstrate higher pre-scores than post-scores at most interventions.

Note that the VAS is measured in 0 to 100 mm. All other scales are 0 to 10 for their minimum and maximum values.

**Table 4.** First intervention–estimated marginal means (least square means) and confidence intervals between music and control groups for Numeric Rating Scale, N = 41.

Group	Marginal means (SE)	*df*	Confidence interval
Control pre-intervention	3.67 (0.52)	35	2.61–4.72
Control post-intervention	3.29 (0.53)	35	2.22–4.36
Overall control	3.48 (0.48)	35	2.50–4.46
Music pre-intervention	5.05 (0.53)	35	3.97–6.13
Music post-intervention	3.34 (0.56)	35	2.21–4.47
Overall music	4.20 (0.50)	35	3.19–5.20

Figure 4 displays this graphically.

Patient interviews were conducted after ICU discharge with study participants who completed either the music or standard care interventions to assess patients’ ICU and study experiences. Three patients in the music group were not interviewed either because they refused or had been taken off the study. Seventeen music interviews were completed and analyzed qualitatively for common themes. Two researcher assistants and a nurse very experienced in qualitative techniques (G.R.W.) independently reviewed the transcribed interviews. Most of the interviews were performed by the principal investigator (N.A.). None of these reviewers had participated in the original interviews. Table 5 describes the major themes and illustrative quotes for each theme. Many patients in the music group (12 out of 17 patients) described the music listening effects as “soothing” or helped them “relax.” Some reported that music helped them to fall asleep (n = 9) and avoid focusing on pain and other stressors. Although none of the patients used the phrase “zone out,” 4 patients described that the music helped them as described in this quote, “the music shut down the outside environment.” Although directly asked about pain management, 2 patients specifically stated that the music reduced pain. Eight patients talked about the environment of the ICU. Patients discussed the noise and the frequency of being checked to “hearing stuff and you just instantly panic.” Three patients complained that the ICU was “noisy” due to beeping monitors, staff conversations, and frequent interruptions. Four patients mentioned the ICU and lack or disrupted sleep. Some patients (n = 6) described how the headphones felt and noted that the noise-canceling headphones blocked ambient noise. For some, this was a benefit, but others felt more anxious because they were unaware of their surroundings during the music listening interventions. Furthermore, some patients (n = 3) did not like the predetermined music selection. These patients said that they would have preferred a different genre of music with lyrics. Many music group participants (n = 11) said that they would use music listening again if given the option.

**Table 5.** Illustrative quotes.

Theme	Quotes
***Environment study effects***	
Environment (the setting of the critical care unit)	“And especially in the ICU there’s always something going on so I think that would really calm people down if they knew they weren’t in danger you know because sometimes you start hearing that stuff and you just instantly panic”
Sleep (as it relates to the setting)	“ICU is not a place to sleep or rest. Every hour on the hour a machine is going off on my arm or they’re coming in to give me something”
***Music study effects***	
Decrease stress and anxiety	“I was glad that I . . . randomized to the music group because it was really useful that . . . key period when I got out of the ICU and I was stressed out um and I got to de-stress using the . . . music shuts down the outside environment was really nice”
Pain reduction	“I think that music helped control the pain you know . . .” “The music in the ICU there’s limited options for how many um times people come in and that kind of stuff so . . . those blocks of time I was listening to the music . . . that first time it was an absolutely a pain reduction response”
Relax	“But I know the stress of not knowing the fear of not knowing and everything they uh started the music sessions and stuff and . . . it really eased a lot cause I was so tired being so stressed that day and suspenseful and stressed and cause I’m not knowing what’s going to hurt and happen and everything so uh it just I don’t know it just relaxed me. It relaxed me so much and I could just lay and then I found I was sounds asleep you know and I was sleeping I was thinking back of my head this stuff probably ain’t going to work or something but it really did work and I told my husband I said this stuff is really good idea I said because it did it really worked good you know” “Oh I just thought it was very peaceful, relaxing um I think it’s a good idea”
Sleep	“I probably got through 15 minutes of the music and it just put me out and I don’t usually go to sleep listening to music, I don’t usually try to go to sleep listening to music and it was just so calming and sedative” “But the music was great, yeah it helped me go to sleep and as long as I am asleep I don’t feel pain”
Zone out	“Well the music I found to [be] very soothing. And I was very pleased with the selections of music and I think it helped the ICU experience because there’s always noise everywhere and it helped to screen out some of the normal noise of an IC unit”
Negative music experience	“Not only the providers but you know my husband, whomever, I needed to be able to um talk to them you know if somebody was going to say okay on a level on 1-10 what’s your pain level well you know you need to be able to hear that” “I would fall asleep and it would be awhile before I pushed the button again which I had been pressing fairly regularly which I hadn’t been doing and know it was 50 minutes of music and I don’t know how long I fell asleep for but I missed some of the button pushes to keep me out of pain and I’d wake up because I hadn’t pushed the button, I would be in more pain”
***Preferred method of delivery***	
Interruption—lack of control	“And then other times it wasn’t a time factor as far as length but just bad timing . . . Like I would put it on when I thought I had nothing going on . . . and everybody would want to say something and you stop and you k now so if I could control that a little bit better”
Preferred type of music	“Yeah there was a point you know a point where I just can’t listen to that again I’ve heard it three times and as it’s I want to participate I’m like not now” “Um if you could do some probably soft, instrumental type stuff. You know . . . than all the brook-y sounds . . . and the birds and stuff like that. So even though that’s a good place to be, also if you had the instruments that were just playing soft stuff”
Timing of music	“Yeah and thinking about that you k-now remember how much they try to put the pain control in the hands of the patient it would be nice to put the music control in the hands of the patient then I could do it as much as I want and yeah” “I would’ve liked to have it the whole time I was in there”
**Headphones**	
Negative	“That’s why I just couldn’t do the earphones because it was one more thing to think about to put on my head to even hear any kind of music. I wanted to know what people were saying how it was being said if somebody asked me how I felt or whatever I needed something so that I could hear what was going on” “Maybe probably if it was just a low where you can just barely hear it you know like doctor’s office you know dentist’s office you know whatever you seem then that would probably be different because you’re not confined you’re not obstructing whatever else is going around you know can be alert of what I going around”
Positive	“I just um to be honest with you I was just listening to the music and I just I don’t remember anything around me plus the headsets are so big and they’re comfortable but they were so big that it would drown out any sounds around and you were just listening totally to the sound” “And because it is when you listen to music on headphones it’s much more you’re much more able to focus on it if it’s in the room then there’s still the beeping and the dinging and whatever so if it’s if the headphones are on I can really that stuff goes away so I think the headphones are critical”

These quotes were captured from the interviews recorded after the patient’s intensive care unit stay. The main themes are displayed in this table.

Besides the 3 participants in the music group who asked to discontinue study participation after starting the first intervention, 2 other participants did not wish to continue listening to the selection after 2 interventions. These participants cited dislike of “confining” headphones and refusal to listen to music as reasons for stopping. Two patients out of 5 had pain scores of 10 when they requested to stop the music listening. No participants in the control/quiet resting group requested to stop the interventions at any time. One patient in the control group inadvertently listened to music before the last intervention. This additional intervention was not entered into the analysis. Three patients were only offered the intervention 3 times a day when the protocol first started. One patient refused to listen after starting the first music listening period as reported above.

## Discussion

Music listening had no significant effect on opioid use between control and experimental groups (Figures 2 and 3). Although there was no statistical difference, data showed that the music group participants used more total intravenous PCA opioids than control subjects throughout the study. In the group of patients with epidurals, music listening subjects used more fentanyl within the first 24 hours of ICU stay, but less within the second 24-hour period (Figure 3). Again, this difference was not significant.

In addition, the differences in pain, distress, and anxiety scores did not differ significantly between groups. Table 3 examines the model estimated mean differences between the NRS, VAS, and ETs. The differences between pre- and postscores were very small.

Studies examining the effects of music have had conflicting results. Some studies in postoperative surgical patients have demonstrated significant reductions in pain and/or anxiety after music listening.^7,32–36^ Many other studies examining the effects of music listening during various procedures, such as colonoscopy, also demonstrate this positive effect of music on pain and/or anxiety.^37–39^ It is important to note that the insignificant correlation between music listening and anxiety scores in this study might be attributed to our small sample size relative to similar studies. However, to date, the effect of music listening on pain is small, subjective, and inconsistent.^13^ In the Cochrane review of 51 studies examining the effect of music on pain, only 14 studies were included that examined postoperative pain. Among these 14 studies, only a small percentage examined the effect of music on pain in a critical care environment.^13^ Recently, a meta-analysis examining music in pediatric surgery patients (n = 196) recommended music as a useful intervention for clinical use.^40^ Although music demonstrated a significant effect in the abovementioned studies, there are other examples in the literature that conflict with these studies, demonstrating conflicting or no significant differences between music listening interventions and standard care in reducing sedative or opioid use.^41–43^ Many studies used only a single intervention and shorter intervention lengths of only 20 or 30 minutes,^6,44^ whereas this study implemented approximately 50-minute music listening or quiet resting periods. One recent study that demonstrated a significant reduction in anxiety allowed the ventilated patient to listen to music as often as requested.^45^ This ventilator study demonstrated a significant decrease in anxiety in intubated patients. Interestingly, the mean minutes per day for the ventilated patients who listened to music were 79.8 (SD: 126) with a median time of 12 minutes and a range of 0 to 796 minutes.^45^ We chose a music selection that was approximately 50 minutes in an attempt to standardize the “dose” of music that was received by each participant. Many studies used 20 to 30 minutes of listening time at varying times during the day or study.^6,33,46–48^ Although we attempted to maximize the music listening “dose” through 50-minute periods, we acknowledge the subjective nature of the music listening experience and recognize a variety of both genres and durations may yield the greatest potential benefit.

The significant finding identified in this study was a significant interaction between the pre- and post-NRS at intervention time point 1 (Figure 4). Using linear mixed modeling, this first intervention was unique from other time points and several factors contributed to this difference. Time point 1 examined pain scores in 37 patients (complete pre- and post-interventions)—one of the largest samples in this study. These measurements, pre-and post-NRS during the first intervention, were recorded shortly after surgery and reflected the unsettled nature of patients being admitted to the ICU from the operating room. Once the patient was admitted to the ICU, they were assessed and started on pain medication with a PCA. The patients began to experience pain relief after opioid administration and music listening. This combination might have contributed to decreased pain in the music group when measured pre- and post-intervention as compared with the control group. A significant interaction effect means that for the 2 groups, namely, music and control, there was a different trajectory before and after the first intervention. The control and music groups reacted differently to the first intervention.

Postoperative pain is linked to numerous, negative health outcomes, including poor wound healing, decreased immune function, increased length of hospital stay, and a prolonged recovery period.^49,50^ The identification and development of an integrative, alternative modality that could decrease patients’ pain and anxiety throughout their recovery from surgery would be important. We hypothesized that music listening would have an effect in reducing pain, distress, and anxiety scores over time. We identified a significant difference in self-reported pain scores before and after the first intervention. No other effect of music listening was identified.

A strong significant correlation was also found between State and Trait Anxiety Inventories, ETs, and GAD-7 questionnaires. The STAI is a validated tool that has been the first choice of many researchers for quantifying anxiety; however, both the shorter and less-intensive GAD-7 and the Distress and Anxiety ETs were better suited for critical care and other clinical environments. This finding supports the use of the ETs in the critical care setting based on less burden to respondent and provider alike.

The major limitations of this study include not only decreased sample size, but also lack of choice of music type, duration of listening, and lack of an objective measure of pain. The most important limitation in this study was sample size. The study was stopped after a discovery that the PCA device could not accurately deliver 2 mL or fewer per hour in the continuous mode. Recognizing that this alert would significantly change prescribers’ practices, the study was ended. Prior to this alert, only 1 patient in this study had been ordered a 2 mL/h or fewer continuous infusion. This patient received that rate for approximately 3 hours. In addition, the sample size was decreased as many (18) failed the randomization criteria postoperatively. Despite this small sample size and the exploratory nature of the study, interesting trends were identified. In addition, the small sample size prevented the stratification of results to surgery type. It would be important in future work to examine either one type of surgery or to randomize on surgery type such as abdominal or thoracic.

There is little agreement in the literature on the optimum time adults should listen to music to have the greatest effect. However, the recent study by Chlan et al^45^ suggests that participants should be allowed to listen to music unobstructed by the study parameters. Another limitation became clear as more participants were interviewed after listening to the music selected by the study team. An alternative design may include querying patients prior to surgery to select music genre.

Vital signs including blood pressure and heart rate could be used as objective measures of pain. However, in this study, because of surgery and anesthesia, these signs would not be appropriate as many patients may experience complications such as hypovolemia. Recently, investigators have been examining skin conductance as an objective measure for pain.^51^ With more research, this might be a useful tool to measure pain.

The 50-minute “quiet resting period” may have affected the control group as this was not part of the standard postoperative care. Furthermore, despite frequent education and reminders, the nursing staff did not always complete all study interventions. In addition, many of the patients transferred out of the ICU sooner than predicted.

Another important limitation was the subjective nature of music listening and its potentially small effect on opiate use. A modality such as music listening may be more appropriately measured in a qualitative way rather than through a single outcome measure such as opiate use. “Music-induced analgesia,” as it was termed in one recent study of the effects of music on MRI findings, explained the intricate neural physiologic response caused by listening to music while invoking a painful stimulus.^52^ This study underscores the complexity of music’s effects and further emphasizes that additional research is needed to understand the influence of music on pain.

One of the more surprising qualitative findings of this study was that music listening interventions inspired polarizing opinions and subjective experiences. Some patients had positive responses to music listening, but many were bothered by the music and/or the headphones. Although patients reported that music was a distractor, some patients disliked feeling disconnected and unaware of ambient noises. This response to wearing headphones, increased anxiety due to a reduction in ambient noise, has not previously been described in the ICU environment. In fact, just the opposite has been reported. In a recent publication, ventilated patients expressed that noise-canceling headphones helped them sleep and block out the noise of the unit when they were awake, therefore, decreasing anxiety.^53^ Currently, many critical care environmental studies examine how to decrease ambient noise without taking into account the patient’s need to be able to hear what is happening in the immediate surroundings. These studies should consider that patients desire auditory awareness in this setting. Therefore, the use of headphones in the ICU requires further study. Another issue points to the subjectivity of music. It is possible that allowing patients to choose their own music from a library of instrumental music with slow tempo, major key tonality, and other soothing attributes, such as nature sounds, would have made patients’ opinions more universally positive toward the music listening experience. Additional studies focused on the ICU environment, music, and its effect on critical care patients should be planned.

### Clinical implications

Music is an integrative, complementary modality that could provide a safe and simple intervention to critical care patients. In this study, some patients really enjoyed the music and felt that it was able to assist them with remaining calm and comfortable after their surgery. Because music listening is such a subjective experience, a larger sample size would be important to demonstrate significance and would be important in planning future studies. At the present time, offering patients music as part of their standard postoperative care is not done in most critical care units. More work is needed to characterize music listening as an intervention. However, this study suggests that this nonmedicinal approach to symptom management could have a place in this environment.

## Supplementary Material

Supplementary material
