# Supplementary material for: Music Listening Among Postoperative Patients in the Intensive Care Unit: A Randomized Controlled Trial with Mixed-Methods Analysis
Source: Integr Med Insights. 2017 Jul 20;12:1178633717716455. doi: 10.1177/1178633717716455 (PMC5588801; doi:10.1177/1178633717716455)
Supplement: Supplementary material [file IMI716455_Supplementary_Material_REV1629.pdf]

Supplementary Information

Appendix A

Emotional Thermometers
Pain Scales

Appendix B
Equianalgesic Calculation

Appendix C
Interview guide for music group

Appendix A

Scales

Distress and Anxiety Thermometers

Please mark how distressed you feel now. With 0 equal to no distress to 10 equal to extreme distress.

Emotion Thermometers (ET)

Instructions

Please mark the number (0-10) that best describes how much emotional upset you are experiencing.

	1. Distress		2. Anxiety	

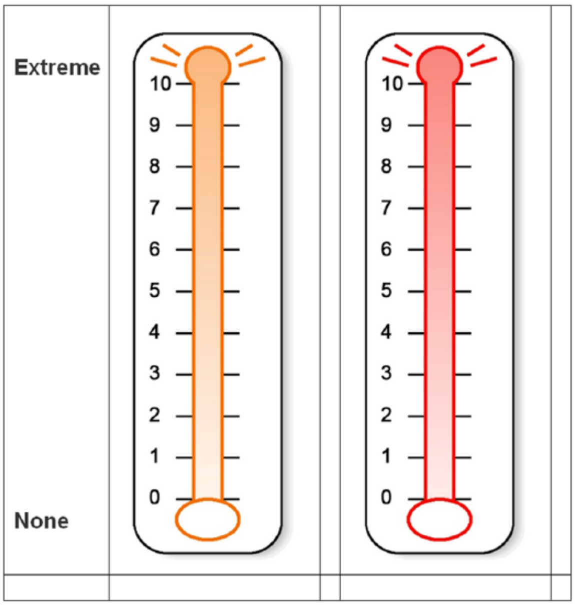

Adapted from the NCCN Distress Thermometer. Alex Mitchell ©

Used with permission.

Appendix A

Scales

Numerical Rating Scale

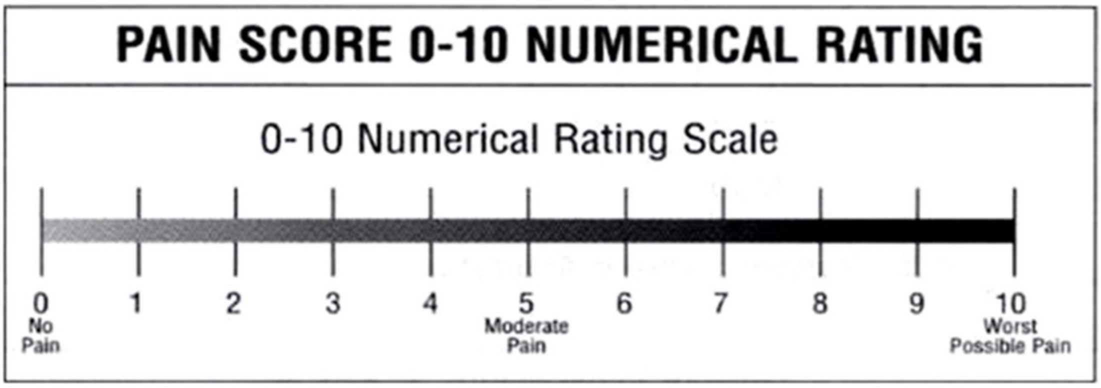

Visual Analogue Scale

Place an X on the line below to show your pain:

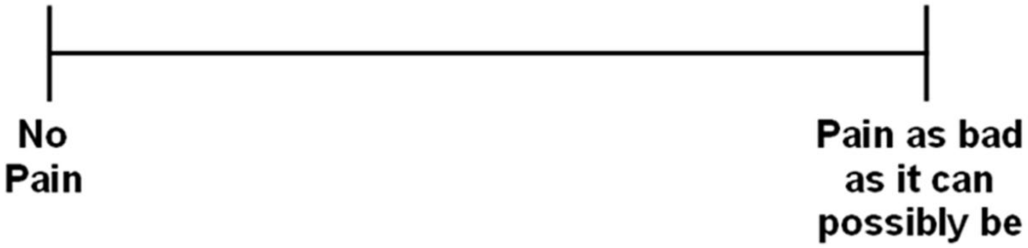

(Note: Not to scale)

Appendix B

Equianalgesic table and conversions [26]

Drug	Intravenous dose (mg)
**Morphine**	10
**Fentanyl**	0.1
**Hydromorphone**	1.5

Intravenous PCA

All non-morphine drugs (fentanyl and hydromorphone) were converted to morphine using the ratios in the above table.

Epidural PCA

No conversion was used. All epidurals contained fentanyl. Fentanyl doses were reported.

Appendix C

Interview Questions

Semi-structured Interview guide
Examples of questions asked of music group participants
What did you think about listening to music while in the ICU?
How did it make you feel?
(Interviewer be careful not to add your own description of the music-let the patient tell you)
Can you describe whether it helped you?
What did you think of the music selections?
What did you think of the length of time you listened to the music? How did you feel about listening to the music three (four) times a day? What did you think of the length of time you listened to the music?
Do you think the length and frequency were appropriate? Describe.
Have you ever used meditation, music listening, massage, or other methods of relaxation in the past? If so what method? And did you find it helpful?
Would you use music listening again while in the hospital?
Do you have any recommendation regarding the music listening program?
